# Patterns of richness of freshwater mollusks from Chile: predictions of its distribution based on null models

**DOI:** 10.7717/peerj.7097

**Published:** 2019-07-05

**Authors:** Carmen Gloria Fuentealba Jara, Reinaldo Rivera, Cristian Franco, Ricardo Figueroa, Victor Faúndez

**Affiliations:** 1 Instituto de Ciencias Naturales, Universidad de las Américas, Concepción, Chile; 2 Laboratorio de Ecología Evolutiva y Filoinformática, Departamento de Zoología, Facultad de Ciencias Naturales y Oceanográficas. Universidad de Concepción, Concepción, Chile; 3 Departamento de Geofísica, Facultad de Ciencias Físicas y Matemáticas, Universidad de Concepción, Concepción, Chile, Chile; 4 Facultad de Ciencias Ambientales y Centro EULA-Chile, Universidad de Concepción, Concepción, Chile; 5 Departamento de Medio Ambiente y Energía. Laboratorio de Genómica y Biotecnología Aplicada, Universidad Católica de la Santísima Concepción, Concepción, Chile

**Keywords:** Freshwater mollusks, Macroecology, Mid-domain null, Rapoport, Source-sink dynamic, Freshwater molluscs

## Abstract

The freshwater mussels from Chile are characterized by a high percentage of endemism and a fragmented latitudinal diversity, which has been attributed to the features and geomorphological history sculpted by the hydrographic basins. In this work, a set of hypothesis under a macroecological approach is addressed, with the aim to explore environmental, topographic and hydrological factors that define the latitudinal distribution of this mussel group. In order to achieve this goal, Rapoport’s rule, geometrics limits and co-ocurrence were evaluated. In addition, we analyze the source and sink hypotheses through the nested analysis. We observed a noticeable mid-domain effect (MDE), where a major richness than expected was randomly observed between 40 and 41°S. The results revealed that the distribution pattern was not concordant with Rapoport’s rule (*r* = 0.123; *p* = 0.128). Regarding to historical dynamic of the distribution, the results show a significant nestedness pattern, suggesting a source-sink dynamic, that is, poorer communities are a subset of richer communities in species. According to the co-occurrence analysis, an aggregate pattern existed, suggesting potential regulatory mechanisms. The specific richness pattern is explained by the variable seasonality of the temperature with a variance percentage explained of 35%. The full model indicated that variables which characterize the heterogeneity of habitat (i.e. range, Shannon), water availability (i.e., precipitation, density of water bodies) and topography (i.e., altitude area available) jointly explain 40% of the variability of the observed richness. This study shows that the geographical distribution of mollusc richness is mainly explained by mainly climatic and topographical environmental components, as well as by the source-sink dynamics.

## Introduction

One of the characteristics of biodiversity is that it is not homogeneously distributed. This is why spatial patterns of diversity and the processes associated to these have become a subject of study for several decades, at different spatial scales and approaches (Koleff et al., 2008). At a macro geographical scale, it has been suggested that phylogenetic diversity and the specific richness of Chile is lower, unlike the Nearctic region (Domínguez & Fernández, 2009), as such some explanatory mechanisms have been put forward, with Rapoport’s rule (Stevens, 1989), standing out. This suggests the existence of an original latitudinal set and decreasing gradient from North to South, as a correlation of the latitudinal extension of the geographic range size of a species and the latitude (Stevens, 1989). The size of the geographic range increases on the maximum limit of a physical gradient (Hernández, Moreno & Rozbaczylo, 2005) which is not attributed to the adaptation of organisms to extreme climatic conditions (Stevens, 1989). Although this mechanism could explain the richness pattern, it has not been evaluated to date in previous studies (Fuentealba, Morrone & Figueroa, 2010); however, it has been written that zones with greater productivity are found in intermediate latitudes, without associating this pattern to any environmental or topographic factor.

An alternative hypothesis to interpret a pattern of greater richness in the center of a domain, where there is no environmental gradient, is the presence of geometric limits or geographical properties, which would determine the spatial distribution of the species (Colwell & Lees, 2000; Jetz & Rahbek, 2002; Grytnes & Vetaas, 2002; Grytnes, 2003), acting as dispersion barriers (Colwell & Lees, 2000; McCain, 2003; Colwell, Rahbek & Gotelli, 2004; Grytnes, 2003). This pattern predicts that the richness spatial gradients would be influenced by geometric restrictions, that is to say, a geographic characteristic that limits the geographic range of the species, acting as a barrier to the dispersion (Grytnes, 2003). This geometric restriction generates the so-called mid-domain effect (MDE) (Colwell & Lees, 2000; Colwell, Rahbek & Gotelli, 2004; McCain, 2004), which is referred to a random accumulation of the geographic ranges of species in the mid-zone of a geographic gradient when the limits of the gradients are hard or define a geographic domain (Zapata, Gaston & Chown, 2003) for instance, the surface of the ocean and the seabed (Moreno et al., 2008).

A tool that allows evaluating this type of hypothesis is the use of a null model (Gotelli & Graves, 1996; Gotelli & McGill, 2006). The use of null models allows assessing the absence of governing factors (i.e., environmental) over the faunistic groups (Gotelli & Graves, 1996), applied to different geographical scales and taxonomical groups (e.g., De Los Ríos, Hauenstein & Acevedo, 2015; Dörr et al., 2012), allowing defining whether the patterns seen are caused by ecological or evolutionary mechanisms or just be chance (Gotelli & Graves, 1996; Vilchis, 2000). The incorporation of the “structureless” null models plays an important role in biodiversity studies which can contribute justifying a determined pattern through a combination of ecological and environmental processes avoiding, in this way, erroneous interpretations of non-existence random patterns (Clarke, Somerfield & Gorley, 2008).

Through Chile, freshwater molluscs represent an interesting model of study, as these present ecological characteristics. Currently, according to the Ministerio del Medio Ambiente (2014), this group is being subjected to drastic ecosystem transformations, as well as the presence of introduced species (Letelier, Ramos & Huaquín, 2007) and the use of pesticides and other chemical substances used in agriculture that affect the viability of freshwater molluscs (Jackson & Jackson, 2011). These factors have led to the modification of the diversity of molluscs. Preliminary aspects regarding their biogeography have been explained by the distribution of the hydrographic system in Chile, suggesting a greater endemism at higher latitudes (Fuentealba, Morrone & Figueroa, 2010). In spite of this information, macroecological studies, destined to explain a determined pattern of richness, have not been explored in this faunistic group (Fuentealba, Morrone & Figueroa, 2010). The following study, based on this background, proposes to describe and explain the richness patterns of freshwater molluscs, evaluating Rapoport’s rule, geometric limitations, co-occurrence patterns and the historical dynamic of the distribution using the sink-source model, as well as ecological mechanisms to explain the patterns of richness of molluscs.

## Materials and Methods

A total of 84 taxa of freshwater molluscs distributed from 18° to 56°S were analyzed. The list of species and their geographical range in degrees is provided in the Supplementary Material 1). The distribution data was obtained from studies carried out by Valdovinos (1989), Valdovinos (2006) and Fuentealba, Morrone & Figueroa (2010). This information was updated by reviewing the new collections deposited in the EULA-Chile Environmental Sciences Center and prospecting performed onsite. A total of 342 records was obtained. After this, these records were mapped into latitudinal bands with one degree of latitude. We make an estimate of the expected richness using the Chao 2 index (Chao et al., 2014) to evaluate the quality of the inventory of species in the latitudinal range under study. The calculations were carried out in the DIVA-GIS 7.5 software (Hijmans et al., 2001). According to the Chao 2 estimator, the total number of species observed in each latitudinal band could be greater than what is currently observed (See Supplementary Material 2). Based on these results, we can indicate that there is indeed a low sampling effort, indicating that the diversity inventory of molluscs is not yet complete, which opens the possibility to direct greater efforts to decrease this bias and have more information on the geographical distribution of freshwater molluscs from Chile.

### Faunistic similarity patterns

To determine the faunistic similarity patterns, a cluster analysis was done on a presence and absence matrix, from which a similarity dendrogram was built, using the Jaccard Index coefficient of similarity based on presence/absence records of the molluscs taxa. For this analysis, we used the unweighted pair/group method with arithmetic averages (UPGMA) as agglomeration algorithm (Sokal & Rohlf, 1995) through PRIMER-6 software (Clarke & Gorley, 2006).

Analysis of the hierarchical cluster and SIMPROF analysis (similarity profile permutation test) were carried out to evaluate the biogeographic zones of freshwater molluscs based on one-degree bands. The SIMPROF permutation test (5% level) was used to determine the clusters with a significant internal structure, using 50,000 permutations (Clarke, Somerfield & Gorley, 2008).

### Rapoport’s rule and geometric restrictions

To evaluate Rapoport’s rule effect, the extension of the latitudinal range was calculated as the difference between the maximum and minimum latitude of the distribution range of each species and the mean calculated as the mean among these values (Gaston, Blackburn & Spicer, 1998). This relation was evaluated by making a regression analysis with a randomization approximation (50,000 random matrices), implemented in the EcoSim 7.71 software (Gotelli & Entsminger, 2004). When the relation between both variables is positive, the hypothesis that the geographical range of the species increases at higher latitudes is upheld, reflecting the possible adaptations of the tolerant species to extreme climatic conditions. Additionally, to explain the distribution of the species richness, the mid-domain effect (MDE), a hypothesis which predicts the existence of a limit or geometric restriction in the distribution of the species, was evaluated. The fact of being a null model, directly excludes any environmental and evolutionary influence over the richness of species (Colwell, Rahbek & Gotelli, 2004). To evaluate the MDE, the specific richness data for each latitudinal band, was compared with a null model, through Monte Carlo simulations of the species richness curves. The simulated curves were based on empirical range sizes within a limit domain using the stochastic analytical model by Colwell & Hurt (1994) and Colwell (1999). In order to do this, we used 50,000 simulations with and without replacement to calculate the amplitude at a 95% confidence interval of the simulated curves (McCain, 2003; McCain, 2004). Sampling without replacement corresponds to a randomization technique, while sampling with replacement corresponds to a bootstrap method (Manly, 1997; McCain, 2004). The analysis was made using the Mid-Domain Null software (McCain, 2004).

### Historical dynamic of the richness distribution

To evaluate the sink-source hypothesis, the degree of nestedness of a presence-absence matrix was estimated, where the columns represent the cells or grids and the rows, the species present in them. After this, it was proceeded to order the matrix according to the total sum by rows and columns, where the common species were placed on the top rows and the cells richest in species were placed on the columns on the left (see Valencia-Pacheco et al., 2011; Vallejos-Garrido et al., 2017). The nestedness metric based on overlap and decreasing fill index (NODF) was used (Almeida-Neto et al., 2008). The values varied from 0 to 100, where the highest values indicate an increase in the degree of nesting (Almeida-Neto et al., 2008; Valencia-Pacheco et al., 2011). The significance was calculated through null models using the Monte Carlo algorithm, contrasting values observed with a random probability distribution. A null model was used with fixed rows and equiprobable columns, where the totals observed by rows are kept, but the totals of the columns varied randomly (Patterson & Atmar, 1986; Gotelli, 2000). This null model conserves the species occurrence frequency, allowing that the specific richness varies equiprobably between the latitudinal bands (Valencia-Pacheco et al., 2011; Vallejos-Garrido et al., 2017). This model was chosen, considering *a priori*, that all the latitudinal bands could be occupied by the species. A total of 50,000 iterations were made to generate the frequency distribution. The nesting analysis was done in the NODF software (Almeida-Neto & Ulrich, 2011). This methodological approximation corresponds to a type of indirect test on the biogeographical hypothesis evaluation in a historical-geographical context, where the metacommunity dynamic is analyzed in a hierarchized or nested spatial system, implicitly incorporating a temporary component on considering large spatial scales (Holling, 1992; Ulrich, Almeida-Neto & Gotelli, 2009). As a result, a nested system will provide, non-random distribution patterns (Connor & Simberloff, 1979; Connor & Simberloff, 1986; Jackson, Somers & Harvey, 1992), where species that are rare in terms of occurrence, will only be present in the groups with greater richness, whereas the most common ones will be present in all the sites (Ulrich, Almeida-Neto & Gotelli, 2009).

### Pattern of co-occurrence of species and regression analysis

To evaluate if the assemblage patterns of species co-occur or are randomly associated, we use the “C score” index (Stone & Roberts, 1990). We calculated a Checkerboard score (“C-score”), which is a quantitative index of occurrence that measures the extent to which species co-occur less frequently than expected by chance (Gotelli, 2000). A community is structured by competition when the C-score is significantly larger than expected by chance (Gotelli, 2000). After we compared co-occurrence patterns with null expectations via simulation (Gotelli, Hart & Ellison, 2015) using the SIM 9 algorithm, which considers the rows and column sums of the matrix are preserved. This means that each random community contains the same number of species as the original community (fixed column), and each species appears with the same frequency as in the original community (fixed row). The analyses were carried out in the Ecosim R package (Gotelli, Hart & Ellison, 2015).

We perform regression models to evaluate climatic, hydrological and topographic predictors as environmental factors that modulate the species richness pattern. Models were generated using the average UV radiation, average annual temperature, temperature seasonality, annual rainfall and seasonality of precipitation as climatic variables, topographic heterogeneity (range, Shannon, average and altitudinal range) as topographic variables and surface of water bodies for each latitudinal band (in Km^2^), and density of water courses as hydrographic variables. The climatic information was obtained from Wordclim database (Hijmans et al., 2005). The solar radiation was obtained from the glUV database (Beckmann et al., 2014), available at http://www.ufz.de/gluv. The variable altitude was obtained from SRTM database 90 m Digital Elevation Database v4.1 (Jarvis et al., 2008), available at https://cgiarcsi.community/data/srtm-90m-digital-elevation-database-v4-1. The topographic heterogeneity was calculated as the altitude range for each 1 degree band. Shannon’s index for topographic heterogeneity is based on textural features of the EVI index (Enhanced Vegetation Index), and was obtained from the EarthEnv database (Tuanmu & Jetz, 2015), available at https://www.earthenv.org/. The descriptive statistics of climatic, hydrological and topographic variables are shown in Table 1).

**Table 1:** Descriptive statistics of the environmental predictors used to explain the richness of molluscs in Chile.

	Annual temperature	Seasonality temperature	Annual precipitation	Seasonality precipitation	Annual UV radiation	Range heterogeneity	Shannon heterogeneity	Altitude (average)	Altitude (range)	Latitudinal band area (Km)	Kernel density river
Min	3.54	215.88	14.26	11.36	1388.52	429.9	10708	231.5	812	2123508	0.005
Max	12.05	429.12	3169.27	130.79	7604.67	6489.89	36323	4316.8	5794	37931470	0.584
Mean	8.57	300.72	949.44	66.56	3924.92	3194.79	26046	1371.7	3208.74	19427610	0.315
Stand. dev	2.72	51.88	871.55	39.56	2005.53	2037.86	9177	1017.1	1481.18	6392766	0.172
Median	9.37	293.35	835.54	77.18	3483.71	3215.85	29941	766.5	2840	18826920	0.326
Q1	5.62	263.95	80.73	20.65	2026.69	1130.51	15386	621.7	1895	15777530	0.211
Q3	11	324.5	1691.65	99.31	6007.97	5488.58	34331	2274.8	4674	23055380	0.479

To evaluate the relationship between species richness and different environmental predictors, we use generalized linear models (GLM) using a Poisson distribution and a “log” link. Since the determination coefficients (R^2^) are not provided by the GLM models, a pseudo-R^2^ was calculated which was obtained through the “rcompanion” package (Mangiafico, 2017). The selection of models was made through the Akaike information criterion (AICc) (Burnham & Anderson, 2002). The analyses were carried out through the MuMin package (Barton, 2017).

All statistical analysis were carried out in R (R Core Team, 2017).

## Results

### Faunistic similarity patterns and geographic ranges

**Figure 1:**
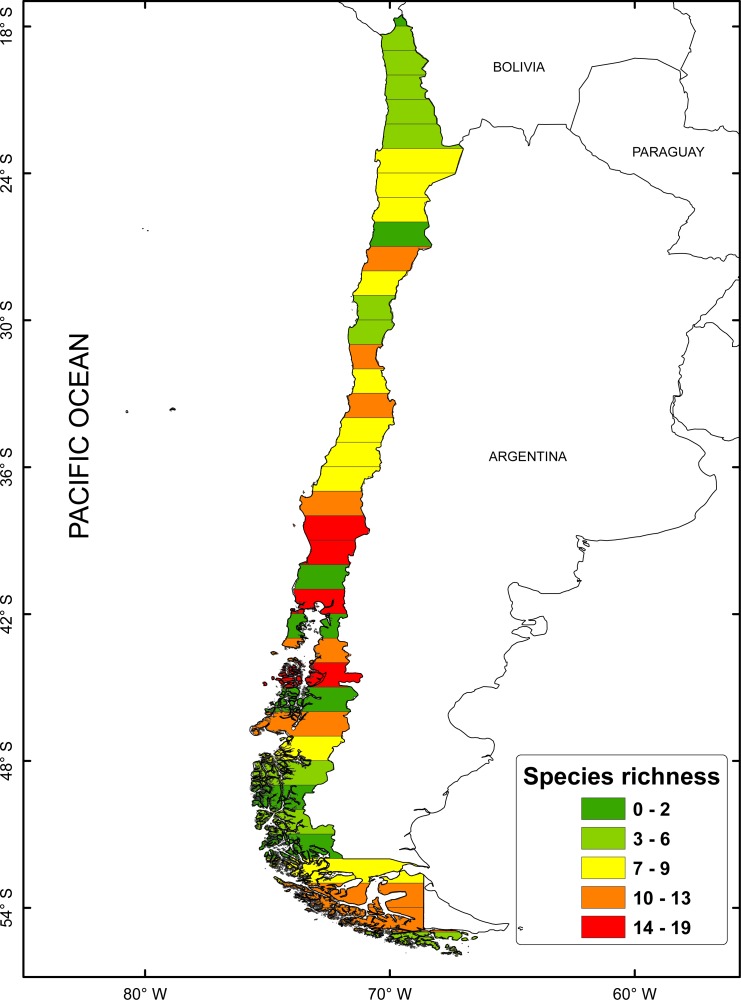
Spatial distribution of species richness for freshwater mollusk in latitudinal bands of 1°.

**Figure 2:**
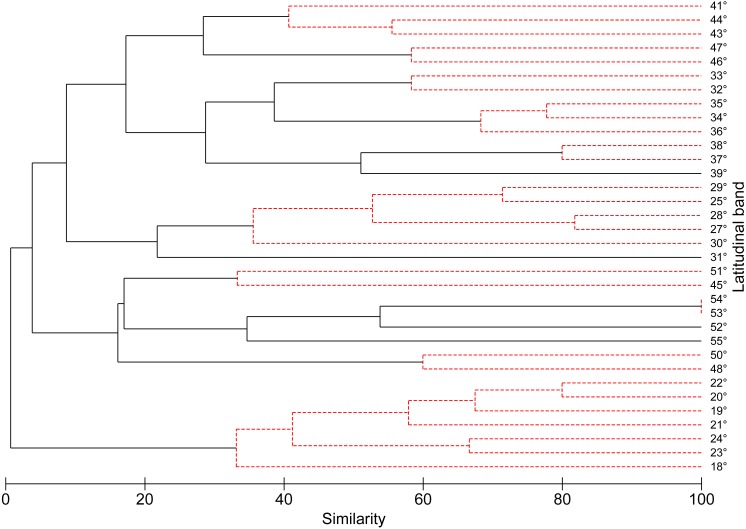
Dendrogram similarity based on the presence or absence of freshwater mollusk species found in 1° latitudinal bands using the Jaccard similarity values and UPGMA as the agglomeration algorithm. The robustness of the dendrogram was established by means of SIMPROF test.

According to the concentration of species by latitudinal band, a greater specific richness is seen between 37° and 42°S, with a maximum richness at 41°S. It is also possible to highlight a lower specific richness at extreme latitudes (Fig. 1). The cluster analysis showed ten groups statistically significant, which correspond to: (1) 18–24°; (2) 25–30°; (3) 32–33°; (4) 34–36°; (5) 37–38°; (6) 41–44°; (7) 45–51°; (8) 46–47°; (9) 48–50°; (10) 53–54° (Fig. 2).

The geographic ranges of freshwater molluscs show a greater frequency of reduced geographical ranges (<2°). In general, the size distribution showed asymmetric bias towards the right (see Supplementary Material 1).

### Effect and mid-domain effect

**Figure 3:**
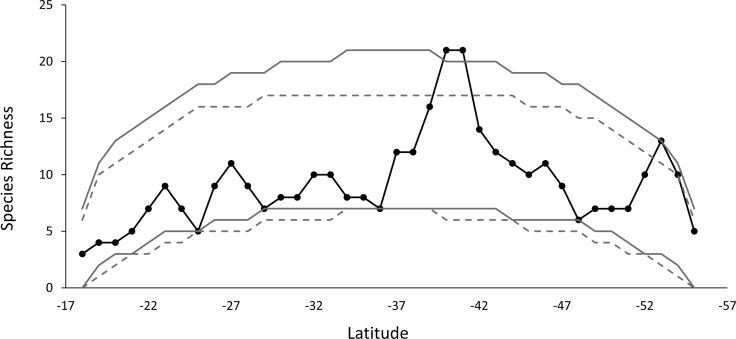
The observed species richness pattern (solid black line), compared to the simulated curves, for the stochastic null model with and without replacement. The gray lines show the 95% prediction curves sampled without replacement (segmented gray lines) and with replacement (solid gray lines).

The species richness curve for each latitudinal band shows a marked MDE. The species diversity pattern observed, compared with the curves simulation at 95% for a stochastic null model without replacement, showed that 98% of the points of diversity are located within the signaled range, a result that is similar to what was obtained for the stochastic null model with replacement. Only one point of diversity does not fit the MDE model, this includes the band located at 40°–41°S (Fig. 3).

### Rapoport’s rule

**Figure 4:**
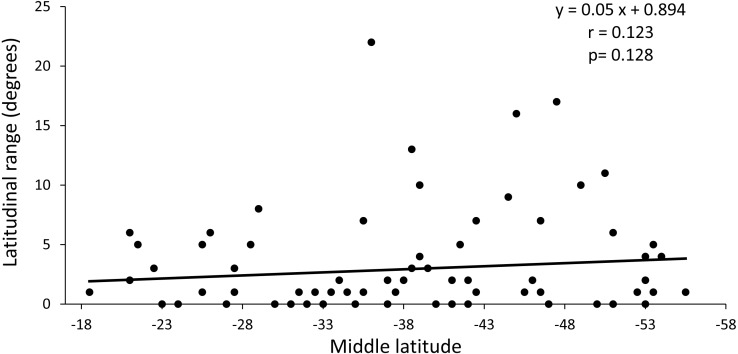
Regression analysis between latitudinal range extent and middle latitude. The value of *p* is the result of the randomization analysis (50,000 permutations) to evaluate the null hypothesis of a slope equal to 0.

The regression analysis between the latitudinal range and the midpoint range showed a non-significant positive slope (*p* = 0.128) and a low value of the correlation coefficient (*r* = 0.014) (Fig. 4). These results indicate that the distribution of species richness is not fitted to Rapoport’s latitudinal rule.

### Nestedness analysis of the richness distribution pattern

Results indicate a significantly nested matrix (except for the BR index), with values lower than expected by chance for the T index, and higher than expected by chance for the NODF index (Table 2). In addition, a significant nestedness was observed between rows (i.e., incidence of species) and columns (i.e., species composition) (Table 2). These results indicate that there is a significant nested pattern, which suggests a sink-source dynamic among the communities.

**Table 2:** Nesting matrix richness of freshwater mollusks in Chile. The T, Temperature, BR, Discrepancy index (Brualdi & Sanderson, 1999), NODF index are shown by columns and rows (95% CI: 95% confidence interval, T, temperature (Atmar & Patterson, 1993).

	Index T	Index BR	Index NODF	NODF Columns	NODF rows
Calculated	14.18	209	14.61	18.71	13.95
Average expected by chance	20.03	212.75	12.55	17.06	11.82
IC (95%)	(17.63–22.41)	(206–219)	(11.8–13.39)	(15.54–18.67)	(11.13–12.61)
*p* value	0.00002	0.1499	0.00004	0.0225	0.00004

### Patterns of species co-occurrence

The simulations of the null co-occurrence model indicated aggregate patterns, indicating that the species tend to be grouped greater than expected by chance (Table 3), indicating the existence of possible ecological mechanisms that underlie this pattern. The histogram of the simulated values and observed value is shown in Supplementary Material 3.

**Table 3:** Results of null model co-occurrence species for total species number.

Algoritm	Observed index	Mean index	Standard effect size	Variance	*p*
Sim9 (RowSums = Fixed; ColSums = Fixed)	7.992	7.4931	11.23	0.00198	<0.001

### Climatic, hydrographic and topographic factors that explain the mollusc richness patterns

The environmental factors that explain the mollusc richness patterns indicate that variables related to environmental energy, such as seasonality temperature, showed a significant relation with richness, explaining 35% of the variability. Another important variable was the average altitude with 28% of the variance explained (Table 4). The other environmental, topographic and hydrographic predictors showed a low percentage of variance explained (Table 4). The full model, that is, with significant climatological, hydrographic and topographical predictors, explains 40% of the variability of specific richness. In this model, the variables related to topography and water availability are the most important according to the value of the estimated coefficient (Table 5).

**Table 4:** Regression coefficients and comparison between the combinations of predictors to explain the distribution of mollusk species richness. The best model is in bold.

Predictor	df	logLik	AICc	ΔAIC	pseudo-R^2^	*P* value
**Seasonality temperature**	**2**	**−134.69**	**273.718**	**0**	**0.35**	**<0.05**
Altitude (average)	2	−136.72	277.782	4.064	0.28	<0.05
Latitudinal band area (Km^2^)	2	−137.93	280.198	6.480	0.24	<0.05
Shannon heterogeneity	2	−139.18	282.700	8.982	0.19	<0.05
Kernel density river	2	−139.85	284.03	10.312	0.16	<0.05
Annual precipitation	2	−140.17	284.681	10.963	0.14	<0.05
Range heterogeneity	2	−140.44	285.218	11.501	0.13	<0.05
Water bodies Area	2	−140.49	285.318	11.600	0.13	<0.05
Average UV radiation	2	−141.42	287.168	13.451	0.09	<0.05
Altitude (range)	2	−142.03	288.391	14.673	0.06	<0.05
Annual temperature	2	−142.18	288.700	14.982	0.05	<0.05
Seasonality precipitation	2	−143.20	290.723	17.005	0.00	n.s.

**Notes.**

n.s. not significant

**Table 5:** Result of full GLM model obtained using a stepwise regression procedure of species richness of freshwater molluscs on environmental variables. This model explained 40% of the variation in the species richness.

	Estimate	Std. Error	z value	*p*
Intercept	25.414	10.088	2.51	0.012
Shannon heterogeneity	−3.166	1.402	−2.25	0.024
Range heterogeneity	2.300	0.721	3.19	0.001
Latitudinal band area	0.168	0.052	3.19	0.001
Annual precipitation	−0.681	0.181	−3.75	<0.001
Altitude (average)	−1.110	0.223	−4.96	<0.001
Kernel density river	4.404	0.786	5.60	<0.001
AIC	241.55			
Pseudo-R^2^	0.4			

## Discussion

The latitudinal climatic diversity of Chile allows the differentiation of a great variety of land and aquatic ecosystems (Parra et al., 2002), so it makes studies about richness and distribution an attractive component along this strip of land. Previous studies based on latitudinal diversity have set out that the layout of the water system is determining in the endemism patterns of freshwater molluscs (Fuentealba, Morrone & Figueroa, 2010), distributed in five macrozones, according to the hydrographic systems proposed by Niemeyer & Cereceda (1984). The cluster analysis, in relation to the richness layout, did not allow validating the hierarchical hypothesis about the distribution in macrozones, and structured spatial similarity patterns, following the characteristics of the hydrographic systems, described by Niemeyer & Cereceda (1984). In regard to the obtained results, 10 groups of molluscs freshwater, those are grouped according to the latitudinal gradient. These groups, show an increase in the richness of species between 37° and 42°S, with reduction at the extreme latitudes of the latitudinal range (18° to 24 °C and between 42° to 56°S). This result differs from what has been reported previously by Fuentealba, Morrone & Figueroa (2010), where the greatest richness was recorded between 33°–44°S, difference which can be attributed to the effect produced by the variation of the scale used (Casagranda, Roig-Juñent & Szumik, 2009). However, it is worth highlighting that the greatest richness observed, would be included within the 35°S and 43°S ranges, previously suggested as hotspots of freshwater invertebrates (Soto & Zúñiga, 1991; Valdovinos, 2008; Pérez-Losada et al., 2002) and as lacustrine zooplankton diversity (Soto & Zúñiga, 1991). These last authors also suggest that, from 38°S to 42°S, the richness of freshwater species is affected by a distributional break and a reduction of the richness towards the south, a latitudinal pattern related to continental breakup events and glacial history.

### Causal mechanisms of latitudinal richness patterns

Rapoport’s rule is not supported by our results because there was no increase in diversity towards the poles, as has been recorded in marine molluscs (Valdovinos, Navarrete & Marquet, 2003). However, there would be a significant MDE as a result of the existence of hard limits at both ends of the continent. In this sense, the geometry of the continent can impose powerful forces which limits the size of the geographic range, being able to counter the ecological and evolutionary processes used to explain Rapoport’s rule effect (Gaston, Blackburn & Spicer, 1998). While there is an increase in diversity towards higher latitudes, pattern reported also for other taxa (Hernández, Moreno & Rozbaczylo, 2005), only one point is greater than expected by chance at −52°S, so in that geographical area there would be a factor or mechanism different from the geometric restriction that would be modulating the richness.

The barriers that limit the dispersion of the organism, have also been preliminary explored in marine taxa (Hernández, Moreno & Rozbaczylo, 2005; Moreno et al., 2008), but not in freshwater organisms. In the MDE case, the first limiting barrier of the spread of freshwater molluscs is located at 18°S, caused by the arid diagonal, causing low water availability basins, most sporadically and under climatic conditions that are highlighted by the extreme aridity caused by the interaction of factors, such as the Pacific Anticyclone, Humboldt Current and elevation of the Andes Mountains (Villagrán & Hinojosa, 1997; Villagrán et al., 1994). Between 41°S and 56°S the continental fragmentation, determining the formation of fjords and glaciers (Camus, 2001), stands out as a barrier to the spreading, where although there is higher water availability, extreme weather conditions for the freshwater fauna stand out. This includes basins with less stability with predominating estuary conditions and the entry of sub-Antarctic waters (Silva, Calvete & Sievers, 1997). Studies made on zooplanktonic fauna from fjords show that the high environmental heterogeneity, limits the colonization of species, intensifying the isolation (Palma, 2006).

### Source–sink dynamics and patterns of co-occurrence

For freshwater molluscs, there is a metacommunity sink-source dynamics, where the zones of greatest specific richness would act as a dispersion source towards other latitudes. According to the results obtained, the latitudinal areas comprising 38–40°S (Araucano Lakes region), would be a source of species, which may colonize extreme latitudes (i.e., sinks). The dynamics of nestedness can be explained by simultaneous balance processes between extinction (Patterson & Atmar, 1986; Bruun & Moen, 2003; Whethered & Lawes, 2005) and selective colonization events (Ulrich, Almeida-Neto & Gotelli, 2009; Valencia-Pacheco et al., 2011), especially in fragmented habitats, current situation of our hydrological resources or even their tolerance, depending on environmental filters (Greve et al., 2005; Driscoll, 2008), as a direct consequence of the geomorphological, climatic and historical events that have determined the current condition of the drainage basins (Fuentealba, Morrone & Figueroa, 2010). Regarding the distributional patterns, species that are rare in terms of incidence are only present in the groups with greatest richness, whereas the most common ones are present throughout the entire latitudinal extension (Ulrich, Almeida-Neto & Gotelli, 2009), represented in our study by the geographic extremes of our study area, previously characterized by extreme conditions and that depend of the tolerance-adaptation of the colonizing species. Another important finding was that the patterns of co-occurrence of freshwater molluscs are not random, or in other words, an aggregate pattern, namely, the species tend to co-occur in a greater way than expected by chance, so potential processes that cause this co-occurrence might be due to the similarity in habitat requirements (Gotelli & McCabe, 2002) or existence of positive biotic interactions (Ovaskainen, Hottola & Siitonen, 2010). This aggregation of species can be linked to a large extent to historical dynamics, such as Pleistocene glaciations and postglacial tectonic events that affected the hydrography of Chile (Fuentealba, Morrone & Figueroa, 2010).

Otherwise, contemporary mechanisms recognize temperature as the main predictor of the distribution of the richness of freshwater fish (Habit et al., 2012), corroborating that this climate variable is a powerful descriptor of freshwater fauna, and for molluscs is able to explain by itself 35% of the variability. On the other hand, our regression models with multiple predictors, indicated that the variables of habitat heterogeneity, topography and water availability (see Table 5) revealed a greater variability explained (40%); therefore, the configuration of the landscape (i.e., heterogeneity) (Stein, Gerstner & Kreft, 2014), as well as the availability of habitat and water regimes are efficient modulators of the richness of freshwater molluscs.

## Conclusions

Rapoport’s rule is not supported by our results as a potential mechanism of the mollusc richness pattern. The effect of average domain was adjusted for a large part of the latitudinal domain studied, observing a greater richness than expected by chance between 40 and 41°S. Our results indicate that the observed richness and its maximum occurred between 40 and 41°S, which can be explained by climatic variables, mainly the seasonality temperature, as well as, heterogeneity of the landscape, and water availability. The historical dynamics (source–sink) is another potential mechanism that would explain the pattern of richness observed for the freshwater molluscs of Chile. These results were supported by the analysis of co-occurrence, which also indicated a pattern of aggregation of the species greater than expected by chance, indicating the existence of mechanisms or regulatory factors that generate this grouping. Finally, these different approximations allowed identifying and describing the richness patterns of freshwater molluscs under a macroecological perspective, their current structuring patterns of the geometric limitation type and the historical dynamic of the distribution, currently subjected to drastic ecosystemic transformations.

##  Supplemental Information

10.7717/peerj.7097/supp-1 Supplemental Information 1 List of species and geographic ranges

10.7717/peerj.7097/supp-2 Supplemental Information 2 Estimating species richness by nonparametric estimator Chao2

10.7717/peerj.7097/supp-3 Supplemental Information 3 Histogram of simulated metric values (blue bars) The vertical red line indicates the observed metric for the original data, the pair of vertical long-dash lines indicate the 95% one-tailed cutpoints, and the short-dash lines indicate the 95% two-tailed c.

10.7717/peerj.7097/supp-4 Supplemental Information 4 Script analysis of co-occurrence

10.7717/peerj.7097/supp-5 Supplemental Information 5 Script regression analysis (GLM)

## Additional Information and Declarations

### Data Availability

The following information was supplied regarding data availability: The regression and co-occurrence analysis scripts are available as Supplemental Files. The regression model script and its data without restrictions, the co-occurrence analysis script and the raw data is also available at: Carmen Gloria Fuentealba Jara, Reinaldo Rivera, Cristian Franco, Ricardo Figueroa, & Víctor Faúndez. (2019). PATTERNS OF RICHNESS OF FRESHWATER MOLLUSCS FROM CHILE: PREDICTIONS OF ITS DISTRIBUTION BASED ON NULL MODELS [Data set]. Zenodo. http://doi.org/10.5281/zenodo.3242858.
