# Supplementary figures and images for: Patterns of richness of freshwater mollusks from Chile: predictions of its distribution based on null models
Source: PeerJ. 2019 Jul 5;7:e7097. doi: 10.7717/peerj.7097 (PMC6613532; doi:10.7717/peerj.7097)

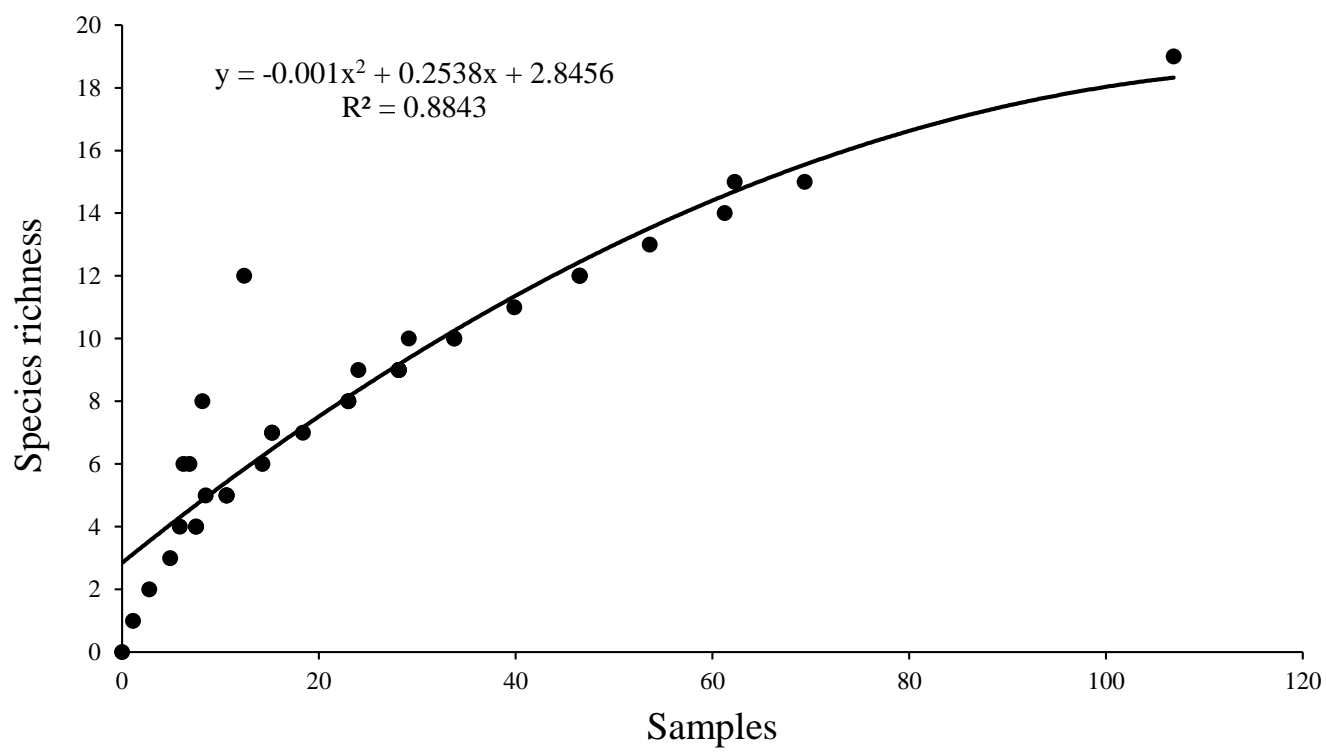

Supplement: Supplemental Information 2 [file peerj-07-7097-s002.pdf]

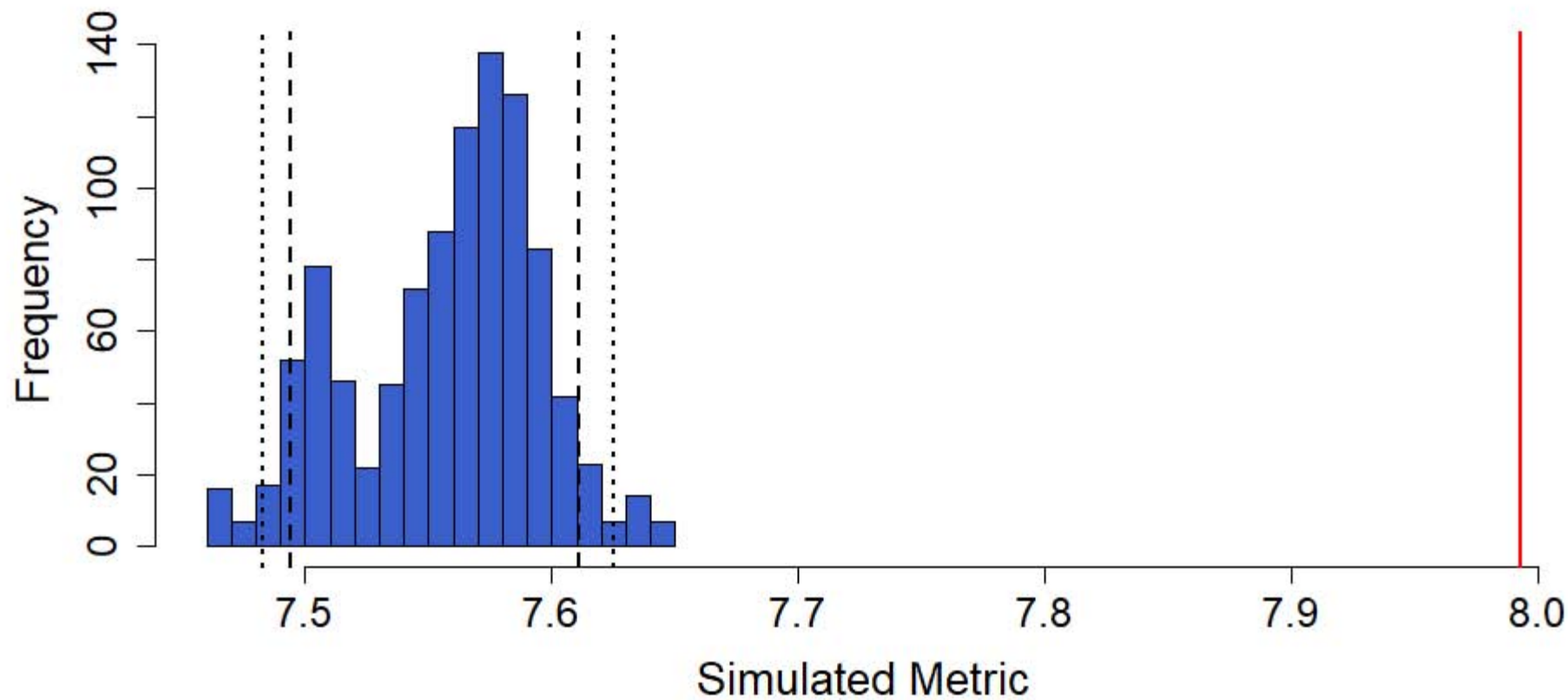

Supplement: Supplemental Information 3 — The vertical red line indicates the observed metric for the original data, the pair of vertical long-dash lines indicate the 95% one-tailed cutpoints, and the short-dash lines indicate the 95% two-tailed c. [file peerj-07-7097-s003.pdf]
